# Pancreatobiliary Adenocarcinoma in a Gastric Duplication Cyst: A Doubly Rare Diagnosis

**DOI:** 10.7759/cureus.16025

**Published:** 2021-06-29

**Authors:** Ana Rolo, Rui Caetano Oliveira, Bárbara Lima, Ana Barbosa, Ilda Faustino

**Affiliations:** 1 Oncology Department, Hospital Senhora da Oliveira, Guimarães, PRT; 2 Pathologic Department, Centro Hospitalar e Universitário de Coimbra, Coimbra, PRT

**Keywords:** case report, gastric duplication cyst, cancer development, adenocarcinoma, pancreatobiliary

## Abstract

Gastric duplication cyst (GDC) is a rare congenital abnormality and the development of malignant transformation in these lesions is even rarer, with only few reported cases worldwide to date. We hereby report an additional case of cancer arising from a GDC in a 54-year-old male. The patient’s chief complaints were abdominal pain and significant weight loss. Computed tomography and endoscopy ultrasonography (EUS) revealed a nodular formation with a cystic component, localized in the great gastric curvature and invading the spleen and left adrenal gland. The biopsy from EUS was inconclusive. After exploratory laparotomy, the patient was submitted to an *en-bloc* resection with partial gastrectomy, splenectomy and left adrenalectomy. Histopathologic examination revealed a cystic mass non-communicating with the gastric wall. Immunohistochemistry staining showed a moderately differentiated pancreatobiliary adenocarcinoma within a duplication cyst with lymphovascular and perineural invasion. The patient was proposed to adjuvant systemic treatment, however, after few months he developed metachronous metastasis. To our knowledge, this is the first case of adenocarcinoma with pancreatobiliary differentiation arising from a gastric duplication cyst.

## Introduction

Gastrointestinal tract duplications (GTD) are a relatively rare anomaly that may occur anywhere along the alimentary tract, from the oral cavity to the rectum, with the ileum being the most frequent locale [1]. Duplications of the stomach are rather rare and most have been reported in children [1,2]. The majority of the gastric duplications are cystic and do not communicate with the lumen of the stomach [3,4]. Malignant transformation arising from a gastric duplication cyst (GDC) is extremely rare, with very few cases reported in the literature to date [5-19]. Adenocarcinoma has been described as the most common histologic type of malignant modification associated with GDC. Malignant transformation increases with patient age [10]. Here, we aim to report an additional case of adenocarcinoma in a GDC, unique by its pancreatobiliary differentiation, together with the review of pertinent literature concerning the development of carcinomas within GDC.

## Case presentation

**Figure 1:**
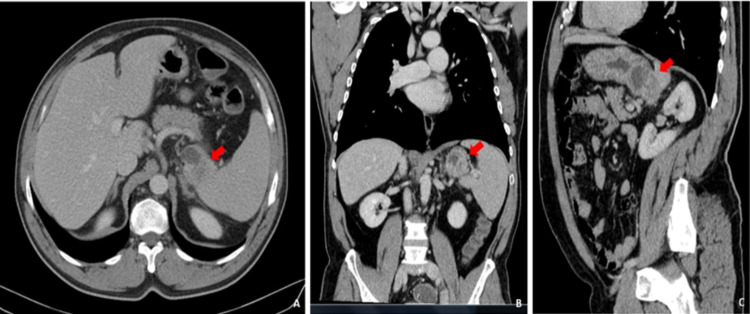
(A) axial, (B) coronal and (C) sagittal CT scans of the chest, abdomen and pelvis demonstrated a mixed mass with posterior tissue component and an anterior component of a cystic nature, localized in the greater gastric curvature, in contact with adjacent structures.

**Figure 2:**
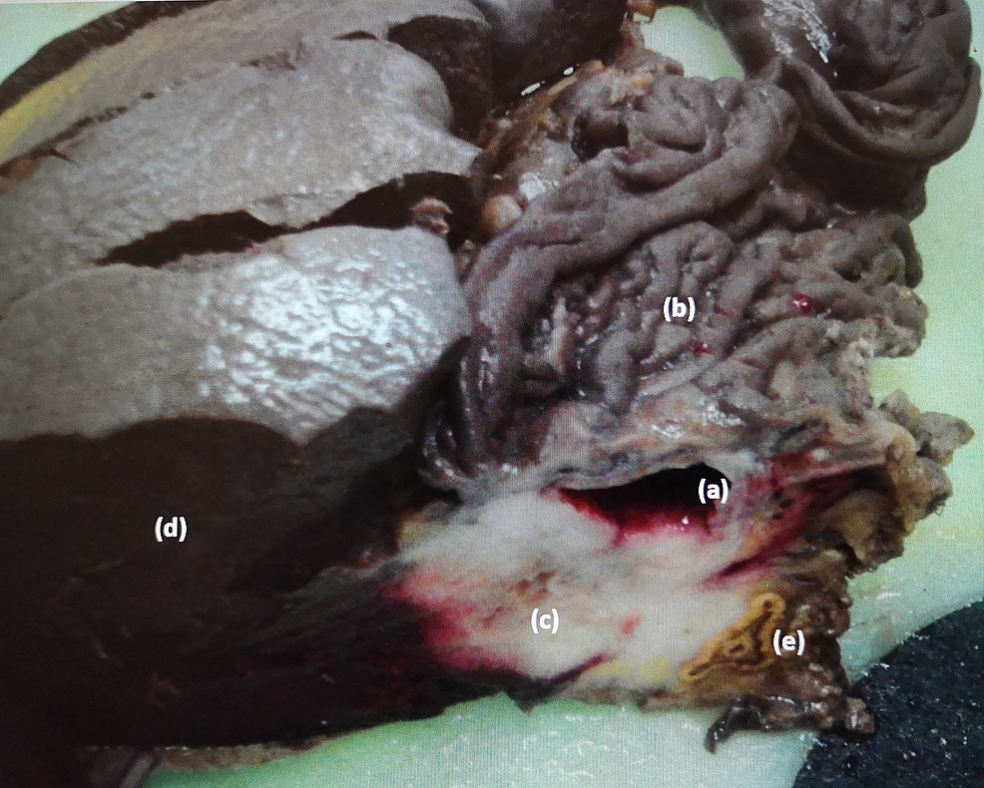
Gross examination of the surgical specimen removed en-bloc showed stomach with a parietal cyst (2 cm) (a), without relation with the gastric mucosa (b). The cyst had ulcerated areas and underlying there was a white and firm lesion of 5 x 1.8 x 1.8 cm (c), which infiltrated the gastric muscular layer, the spleen (d), and the adrenal gland (e). No pancreatic tissue was observed.

**Figure 3:**
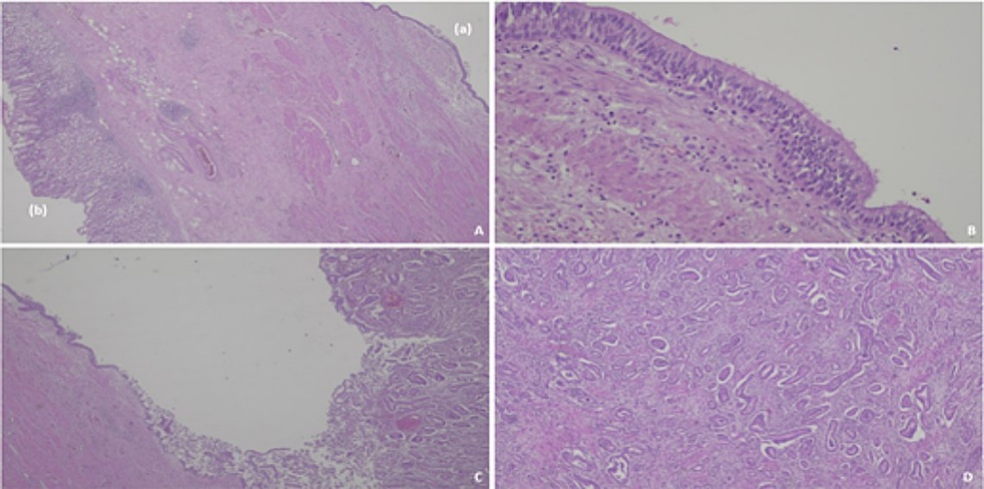
Microscopic appearance by hematoxylin and eosin stains: (A) The GDC cyst (a) was developed in the gastric wall, surrounded by smooth muscle, without connection to the gastric mucosa (b), and was lined by a ciliated pseudostratified epithelium (B), original magnification 20x. Adenocarcinoma developed in the cyst, continuous with the non-neoplastic epithelia (C) and with an invasive component with desmoplastic reaction (D), original magnification 40x. GDC: Gastric duplication cyst

A 54-year-old man presented to the emergency department complaining of a one-month history of non-specific abdominal pain in the left abdominal quadrant and a weight loss of about 10 kilograms in less than six months. Past medical history and physical examination were unremarkable. Laboratory tests were all within the normal range. A computed tomography (CT) scan of the chest, abdomen and pelvis revealed nodular formation (5.1 x 3.9 cm) of mixed nature with a posterior tissue component that captured the contrast product and an anterior component of a cystic nature, localized in the posterior margin of the great gastric curvature, without evidence of lymphadenopathies and no distinct planes with adjacent structures namely with spleen and left adrenal gland (Figure 1A-1C). Upper gastrointestinal endoscopy was normal and biopsy showed superficial chronic gastritis with positivity for Helicobacter pylori. Endoscopy ultrasonography (EUS) was then performed showing a hyperechogenic formation with regular and well-defined limits, apparently unrelated to the gastric wall, with a cystic component. No changes in the morphology and thickness of the different layers of the gastric walls were identified. Biopsy puncture with 19G needle from EUS was done, but fragments obtained were inadequate for diagnosis. The patient underwent an exploratory laparoscopy converted to open surgery when a large tumoral mass adhering to the gastric fundus was found surrounding the spleen and left adrenal gland. He was submitted to an en-bloc resection of the mass with partial gastrectomy, splenectomy and left adrenalectomy. The patient’s postoperative course was uneventful. He was discharged 10 days after surgery. Histopathologic gross examination of the surgical specimen showed stomach with a cystic lesion (2 cm) developed in the gastric wall but not related with the gastric mucosa, with an underlying white and firm tissue (5 x 1.8 x 1.8 cm), with areas of serous content, infiltrating the splenic and left adrenal gland (Figure 2). The lesion developed in the gastric wall, without communication with the gastric mucosa, which had no significant changes. Histologic evaluation revealed that the cyst had ciliated pseudostratified epithelium, underneath with a muscle layer continuous with the gastric muscular layer (Figure 3A, 3B). The cyst exhibited ulcerated areas, where an epithelial malignant neoplasm was evident. The tumor was composed of glands of irregular contours, juxtaposed, widely infiltrative and with marked desmoplastic reaction (Figure 3C, 3D). Phenotypically the tumor displayed strong staining for EMA (MUC1) and CK7, with heterogeneous staining for MUC5AC and MUC6 and no staining for MUC2 and CK20 were registered. Stained slides were observed in a light microscope, Nikon Eclipse 50i, and images obtained using a Nikon-Digital Sight DS-Fi1 camera (Nikon Instruments, Inc., Melville, NY). Eight lymph nodes were isolated without metastatic disease. Lymphovascular and perineural invasion were evident. These aspects were consistent with moderately differentiated pancreatobiliary adenocarcinoma developed in a gastric duplication cyst. The patient was evaluated in multidisciplinary team discussion (MTD) and was proposed for adjuvant chemotherapy (ChT) with modified FOLFIRINOX scheme (5-Fluorouracil 2400 mg/m^2^ 46h continuous infusion; Levofolinate sodium 200 mg/m^2^ D1, Oxaliplatin 85 mg/m^2^ D1, Irinotecan 150 mg/m^2^ D1, 12 cycles, biweekly). At baseline of treatment, tumor markers were negative. Three months after completed ChT, the patient referred an abdominal left pain. The CT scan revealed a nodular lesion with 2.5 x 3 cm adjacent to pancreatic tail and suspect peritoneal implants. The biopsy confirmed that they were metastatic lesions. The patient was discussed again in MTD and was proposed for second-line palliative ChT with nabpaclitaxel (125 mg/m^2^) plus gemcitabine (1000 mg/m^2^), D1-8-15, 28/28 days. After 13 months of treatment, the patient had stable disease in the last response evaluation.

​​​​​​​

## Discussion

GTD are a rare congenital developmental malformation with a reported incidence of about 1 in 4500 live births [16]. These anomalies may occur at any level, from the oral cavity to the rectum, with the ileum being the most common site (33-35%) following by esophagus (20%), colon (13%), jejunum (10%), stomach (7-9%) and duodenum (5%) [1,6]. Although GTD can be found in patients of any age, they are more common in children [1,2].

GDC are a class of GTD, most commonly diagnosed during childhood and very rarely in adults [9,11]. The majority of cases are diagnosed in the pediatric population within the first year of life (67%) and rarely after 12 years of age (less than 25%) [3,6,9]. GDC are twice more prevalent in women and no striking racial or geographic differences have been noted [4,11].

The pathogenesis of these lesions remains poorly defined and several theories have been proposed, like abnormal recanalization during the bowel development (most defended hypothesis), split notochord etiology and remaining diverticula [1,14].

Rowling summarized the essential diagnostic criteria of GDC defined as follows: (a) the wall of the cyst is contiguous with the stomach wall; (b) the cyst is surrounded by smooth muscle, which is continuous with the muscle of the stomach; (c) the cyst wall is lined by typical gastrointestinal mucosa [2]. The present case fulfills the above-mentioned morphological criteria. The mucosal lining of duplication may be histologically similar to the segment of gastrointestinal tract to which it is topographically related or may include lining from other segment of alimentary or respiratory tract, as in our case. Both gastric and pancreatic ectopic tissues can be seen, which are the most common and tend to be the most clinically significant, as patients can develop complications, like bleeding, peptic ulcer disease, fistula, pancreatitis or adenocarcinoma [12].

Although GDC are found anywhere in the stomach, most are located in the greater curvature, as in the present case, followed by posterior wall, lesser curvature, anterior wall and pylorus [11]. Because most cases occur along the greater curvature of the stomach, the cysts are found to compress the adjacent organs such as the pancreas, kidney, spleen, and adrenal gland [16]. Accordingly, the differential diagnosis would include cysts derived from these organs [4]. Two morphologic types of GDC are described, cystic and tubular. More than 80% of gastric duplications are cystic, which means that the lumen lesion is not contiguous with stomach lumen and does not communicate with it. The remaining are tubular with some communication with stomach lumen [1,10].

Gastric duplications typically become symptomatic during childhood presenting either with a palpable mass or by upper intestinal obstruction. Conversely, in adults, they are usually asymptomatic and the diagnosis of GDC is mainly discovered incidentally with ultrasonography, CT scan or gastric endoscopy performed for other indications [4,12]. Those symptomatic present with nonspecific complaints, such as nausea, vomiting, epigastric fullness, imprecise abdominal pain, weight loss, anemia, dysphagia, dyspepsia, or epigastric mass on physical examination [3,9-11]. Complications such as gastrointestinal bleeding, perforation, infection, ulceration, torsion, fistula formation, obstruction, compression or malignant neoplasms arising in these structures could be scenarios of presentation [3,18].

As shown in Table 1, data from all 17 GDC complicated with malignancy cases reported in the literature, including our case, was reviewed and pooled. The incidence rises up with age, given that 71% of the patients are 50 years old or more at diagnosis (median age 54 years and an age range 25-78). Although the malignant transformation of GCD is very rare, it tends to be underestimated due to surgical removal of the cyst after diagnosis in childhood. In opposite, detection of this lesion in adults should raise a strong suspicion of malignancy [13]. Fukumoto et al. suggested that GTD should be recognized as risky lesions of cancer development in patients aged 50 years or above [10]. This review of literature about carcinoma arising from GDC, with many more cases documented in patients 50 years old or above, supported this affirmation. There are more cases of men with GDC complicated with malignancy (65%, n = 11/17), which is in concordance with a higher male predominance of gastric cancer.

**Table 1:** Summary of case studies reporting carcinoma arising in gastric duplication cyst. CF – cisplatin and 5-fluorouracil; ChT – Chemotherapy; EOX – Epirubicin, oxaliplatin, capecitabine; F – female; 5-FU - 5-fluorouracil; FOLFIRINOX – 5-fluorouracil, leucovorin, irinotecan, and oxaliplatin; LND – lymphadenectomy; M – male; NED – No evidence disease; RT – radiation therapy; TS-1: tegafur-gimeracil-oteracil potassium.

Reference	Sex/ age (yr)	Symptoms	Location	Cyst size (cm)	Histology	Clinical Outcome
Mayo et al., 1955 [5]	F/64	Weakness, anorexia, weight loss	Antrum	6.0	Well differentiated glandular carcinoma	Proximal gastrectomy; NED at 12 months
Coit and Mies, 1992 [6]	F/72	Epigastric pain, fullness, weight loss	Antrum	3.2	Papillary mucinous adenocarcinoma	Distal subtotal gastrectomy NED at 72 months
Kuraoka et al., 2004 [7]	M/40	Fever, back pain	Anterior wall of fundus	7.0	Well differentiated adenocarcinoma	Proximal gastrectomy with LND; Liver metastasis at 7 months of surgery, palliative ChT.
Horne et al., 2007 [8]	M/40	Acute abdominal pain, anorexia	Posterior wall of fundus	12.0	Well differentiated neuroendocrine carcinoma	Total gastrectomy, splenectomy, distal pancreatectomy; adjuvant ChT cisplatin+etoposide; Peritoneal metastasis at 14 months after surgery
Barussaud et al., 2008 [9]	F/67	Abdominal pain, weight loss	Antrum	18.0	Mixed adenocarcinoma and squamous cell carcinoma	Total gastrectomy; Liver metastasis at 6 months under adjuvant ChT with CF
Fukumoto et al., 2008 [10]	M/50	Persistent vomiting	Greater curvature of upper gastric corpus and bulbus	2.0, 3.0	Adenocarcinoma	Distal gastrectomy with LND followed by pancreatoduodenectomy; Local recurrence and multiple liver metastases 8 months after; palliative ChT 5-FU and RT; died 14 months after the initial surgery.
Zheng and Jing, 2012 [11]	M/25	Asymptomatic	Greater curvature of the body	8.0	Moderately differentiated tubular carcinoma	Total gastrectomy with LND, adjuvant ChT; NED at 13 months
Kang et al., 2014 [12]	M/56	Asymptomatic	Greater curvature of the body	5.5	Adenocarcinoma	-
Liu et al., 2014 [13]	M/28	Asymptomatic	Gastric body	13.0	Cytology positive to adenocarcinoma	Surgery with cyst lesion ruptured; Peritoneal carcinomatosis 7 months later
Zhu et al., 2015 [14]	M/62	Chronic abdominal pain	Greater curvature of the body	4.0	Well differentiated adenocarcinoma	Total gastrectomy
	M/72	Regurgitation, abdominal distention	Posterior wall of antrum	2.0	Well differentiated adenocarcinoma	Radical resection
Yamasaki et al., 2016 [15]	F/42	Asymptomatic	Greater curvature of the body	10.0	Moderately differentiated adenocarcinoma	Partial Gastrectomy without LND; Peritoneal metastasis 7 months after; oral ChT (TS-1); died 2 months later.
Abdulla et al., 2017 [16]	M/51	Melena	Greater curvature of the body	5.1	Moderately differentiated adenocarcinoma	Neoadjuvant chemotherapy (EOX 6 months) followed by total gastrectomy
Sethi et al., 2018 [17]	M/63	Vomiting, hematemesis	Lesser curvature of stomach near antropyloric region	10.0	Papillary adenocarcinoma	Subtotal gastrectomy with LND, and wedge resection of liver
Chan et al., 2018 [18]	F/57	Non-specific abdominal discomfort	Posterior wall of fundus	5.8	Moderately differentiated adenocarcinoma	Total gastrectomy, distal esophagectomy, and D2 LND; NED 6 months after surgery
Kinugasa et al., 2020 [19]	F/78	Asymptomatic	Lesser curvature of the lower body of the stomach	11.0	Moderately differentiated adenocarcinoma with one lymph node positive	Distal gastrectomy with en-bloc D2 regional LND and Billroth 1 reconstruction (pT3N1M0, IIB); Adjuvant oral ChT TS-1 12 months; NED after 4 years
Present case	M/54	Abdominal pain, weight loss	Greater curvature of the body	5.0	Moderately differentiated pancreatobiliary adenocarcinoma	Partial gastrectomy, splenectomy and left adrenalectomy; ChT 12xFOLFIRINOX Local recurrence and peritoneal metastases 3 months after ChT; 2^nd^ line ChT nabpaclitaxel/gemcitabine

The majority of cases diagnosed with carcinoma were symptomatic with a variable complaint, with abdominal pain and weight loss being the most prevalent. Localization in greater curvature was more frequent but many other locations were registered. Like previous reports [16,18], adenocarcinoma was the most common histologic type of malignancy arising in GDC cases; however, neuroendocrine carcinoma [8] and mixed adenosquamous cell carcinoma [9] have also been reported. To our knowledge, this is the first case of adenocarcinoma with pancreatobiliary differentiation described in these lesions. As mentioned by other reviews [7,12], also in our case the adenocarcinoma found in the cyst cavity invaded the stomach wall, but no adenocarcinoma in situ or precancerous lesions (epithelial dysplasia, atrophic gastritis or intestinal metaplasia) were detected in the mucosa of the stomach, which support the idea that the carcinoma arose from the diagnosed GDC.

The mechanism of malignant transformation of GDC is not clear and many possible explanations have been presented, nevertheless, they are not confirmed. Gastric duplication has been reported to have ectopic gastric or pancreatic mucosa that contain gastric acid and peptic enzymes, which may cause ulceration and perforation. These persistent irritants together with events, such as increase of intracystic pressure and oxygen deficiency in the local microenvironment, may cause chronic inflammation, repeated apoptosis and regeneration of the epithelium that could ultimately lead to the malignant transformation process in the gastric duplication [14].

The pathological differential diagnosis includes pancreatic pseudocyst, mucinous cystadenoma of the pancreas, splenic cysts, gastrointestinal stromal tumor (GIST), neuroendocrine tumor, malignant transformation arising within a gastric teratoma [4,8,11-13,17]. Malignancies arising from duplication cysts are likely to be present at advanced stages because of their unusual symptoms and difficulty of diagnosis [3].

Even if it is difficult to obtain a preoperative diagnosis of GDC, recent imaging studies have helped. Contrast-enhanced CT scan and magnetic resonance imaging (MRI) are useful for initial diagnosis and are important to determine the size, the cystic nature of the lesion and characterize the cystic contents as well as its extent and relation with the adjacent structures [3,4,8]. GDC appear classically as thick-walled cystic lesions with inner lining enhancement and occasional calcifications on both CT and MRI [3,16,20]. However, the wall is sometimes thin, the enhancement of the inner cyst wall is not always demonstrated in the CT [3], as seen in the present case. MRI was not performed in this case, because our patient had a metallic prosthesis on the left hip that contraindicated it. EUS is useful in distinguishing between the intramural and extramural lesions of the stomach and it plays a major role in the diagnosis by showing the inner echogenic mucosal and the outer hypoechoic muscle layers that are typical of a GDC [3,4,16]. The role of fine-needle aspiration (FNA) in GDC remains controversial, because the sensitivity, specificity, and diagnostic accuracy of EUS-FNA biopsy for GDC are not known [20]. Some authors argued that a cytological and histological examination of the cyst by EUS-FNA was necessary to rule out malignancy [4]. However, the potential risk of disseminating the malignant cells along the needle tract and into the peritoneum should always be considered, together with the risk of infection and bleeding [20].

In the present case, tumor markers were not measured at the time of cyst lesion diagnosis, but they were negative at the beginning of adjuvant ChT. In the reported cases of carcinoma within GDC, carcinoembryonic antigen (CEA) was elevated in one [11] and cancer antigen 19-9 (CA 19-9) in four [9,10,15,19] and both were within normal limits in three descriptions [14,17,18]. In one case, elevation of CA 19-9, CEA and CA 125 was noted in a patient presented with disseminated intra-abdominal metastasis seven months after cyst excision with no malignancy [13]. However, there are described cases of elevation of CEA and/or CA 19-9 without evidence of malignancy. To the date, no predictor of the malignant transformation has been established, including the symptoms, size, location, tumor markers or macroscopic findings [14,17].

Preoperative diagnosis is difficult to do. Nonetheless, the definitive diagnose of GDC can only be made by surgical removal and histological examination. In the cases reviewed in Table 1, with the exception of one case in which the carcinoma within GDC was characterized by biopsy and patient was submitted to neoadjuvant chemotherapy [16], all the other cases of carcinoma described in this lesion were made after resection. There is no accepted consensus of management of GDC. Regardless of whether there is or no symptoms, the treatment of choice of GDC is considered the complete surgical removal, in order to avoid the potential complications described above and given the risk of malignant transformation [8,11]. The use of drainage and marsupialization of the cyst has been discouraged as the unprotected mucosa of the cyst will be exposed to a greater volume of gastric contents, placing it at a higher risk of ulceration [16]. When a malignant transformation is suspected total or subtotal gastrectomy with lymphadenectomy should be performed [11,15]. Liu et al. reported a case of a 28-year-old man with metastatic adenocarcinoma with peritoneal carcinomatosis diagnosed seven months after surgery of GDC, in which the cyst ruptured after an unsuccessful attempt to remove the lesion en-bloc [13]. This case highlights the importance of accurate preoperative diagnosis and optimal surgical management for gastric duplication as well as considering the potential existence of malignant transformation during the surgical evaluation of adult patients with GDC.

Due to the rarity of this lesion, there are no guidelines concerning the use of adjuvant therapy. Based on available data of the cases of carcinoma arising from GDC described above, in five patients follow-up data were unavailable [12,14,16,17]. Ten patients were submitted only to resection without additional treatments [5-7,10,13-15,17,18]. Three of them presented with the recurrence disease after surgery and around 7/8 months of surveillance. Two patients died from metastatic disease. This suggested that carcinoma within GDC may have a poor prognosis. In the operated patients without any further treatment and no recurrence disease [5,6,18], with exception of one patient with 72 months of follow-up, two others cases have short surveillance time to take any conclusions. Relatively to those submitted to adjuvant ChT, one patient was disease free after 13 months of follow-up [11], one had no evidence of recurrence four years after 12 months of systemic treatment [18] and another patient developed generalized liver metastasis under adjuvant ChT [9], but we have to point that peritoneal carcinomatosis was described upon laparotomy. Different protocols of adjuvant ChT were used, maybe guided by local orientations and tumor differentiation.

In our case, the patient had an adenocarcinoma diagnosed in GDC after resection of preoperative mass study with the higher expression of CK7 and EMA immunostaining compatible with a pancreatobiliary phenotype differentiation. After review of the literature and considering this data, based on the presence of known poor prognostic factors in gastrointestinal malignancy, such as lymphovascular and perineural invasion or insufficient lymph node resected, our patient was proposed, in multidisciplinary team discussion, to do adjuvant chemotherapy with FOLFIRINOX, the standard of care in pancreatic adenocarcinoma in this setting. Unfortunately, three months after completing ChT, peritoneal metastasis was confirmed and following the orientations of metastatic pancreatic cancer, he was proposed to second-line palliative ChT with nabpaclitaxel plus gemcitabine, presenting stable disease after 13 months of treatment.

## Conclusions

GDC is a congenital rare condition that may pose a diagnostic challenge. Even though a panel of imaging modalities is available, it is still difficult to obtain a preoperative diagnosis of GDC and they should be considered when a cystic lesion is found adjoining the gastric wall. GDC may predispose to complications, including malignancy transformation, as in our case, which is an even more rare condition. We herein report the case of a 54-year-old man with invasive pancreatobiliary adenocarcinoma arising from a GDC. To our knowledge, this is the first case reported with this phenotype arising in a GDC and allows us to add information to very limited literature on such tumors. More cases of carcinomas within the GDC must be described to draw definitive conclusions about the tumor’s distinct behavior and its appropriate management.
